# Probiotic fermentation augments the skin anti-photoaging properties of *Agastache rugosa* through up-regulating antioxidant components in UV-B-irradiated HaCaT keratinocytes

**DOI:** 10.1186/s12906-018-2194-9

**Published:** 2018-06-26

**Authors:** Daehyun Shin, Yoonjin Lee, Yu-Hua Huang, Hye-Won Lim, Kyounghee Jang, Dae-Duk Kim, Chang-Jin Lim

**Affiliations:** 1 R & D Center, Cosmocos Corporation, Incheon, 21698 Republic of Korea; 2 College of Pharmacy and Research Institute of Pharmaceutical Sciences, Seoul National University, Seoul, 08826 Republic of Korea; 3 R & D Center, Shebah Biotech Inc., G-Tech Village, Chuncheon, 24398 Republic of Korea; 4 Department of Biochemistry, College of Natural Sciences, Kangwon National University, Chuncheon, 24341 Republic of Korea

**Keywords:** *Agastache rugosa*, Anti-photoaging, Glutathione, HaCaT, Matrix metalloproteinase (MMP), Probiotic fermentation, Reactive oxygen species (ROS), Superoxide dismutase

## Abstract

**Background:**

*Agastache rugosa* (Fisch. & C.A.Mey.) Kuntze (Korean mint) is used to treat diverse types of human disorders in traditional medicine. In recent years, its non-fermented leaf extract (ARE) has been shown to possess protective properties against ultraviolet-B (UV-B) radiation-induced photooxidative stress. The present work aimed to examine whether probiotic bacterial fermentation would potentiate the skin anti-photoaging activity of ARE or not, by comparing the protective properties of ARE and corresponding fermented extract (ARE-F) against UV-B radiation-induced photooxidative stress in HaCaT keratinocytes.

**Methods:**

ARE-F was produced from ARE by the fermentation with *Lactobacillus rhamnosus* HK-9, a type of Gram-positive probiotic bacterial strain. Anti-photoaging activities were evaluated by analyzing reactive oxygen species (ROS), promatrix metalloproteinases (proMMPs), total glutathione (GSH) and total superoxide dismutase (SOD) in UV-B-irradiated HaCaT keratinocytes. Antiradical activity was determined using 2,2-azino-bis(3-ethylbenzothiazoline-6-sulfonic acid) (ABTS) radical scavenging assay.

**Results:**

ARE-F contained higher attenuating activity on the UV-B-induced ROS generation than ARE. Similarly, ARE-F was able to diminish the UV-B-induced proMMP-9 and -2 more effectively than ARE. ARE-F displayed higher tendencies to augment the UV-B-reduced total GSH content and SOD activity than ARE. However, there were no significant difference between ARE and ARE-F in ABTS radical scavenging activities.

**Conclusions:**

The findings suggest that the UV-B radiation-protective activity of ARE is enhanced by probiotic bacterial fermentation, which might improve the therapeutic and cosmetic values of *A. rugosa* leaves.

## Background

Skin aging, concisely defined as an impairment of skin integrity, is classified into the two main types: intrinsic or chronologic aging and extrinsic aging or photoaging. Intrinsic aging comes from the common process of senescence affecting all body organs, whereas photoaging occurs as a consequence of chronic exposure to environmental factors, especially solar ultraviolet (UV) radiation, and is characterized by skin fragility, wrinkle, roughness, sagging, dryness, laxity and hyper-pigmentation [1, 2].

The three types of UV radiation – UV-A, UV-B and UV-C – have different wavelength ranges. Although UV-C radiation (100–280 nm) is extremely damaging to the skin, it is almost completely absorbed by the ozone layer and thus has no adverse effects. Despite being more penetrating deeper in the skin, less energetic UV-A radiation (315–400 nm) has been considered to be less harmful. Recent studies implicate that UV-A radiation can cause skin cancer indirectly via generating highly reactive chemical intermediates, such as free radicals and ROS, which in turn can damage DNA [3]. UV-B radiation (280–315 nm) constitutes only 5% of the total solar UV energy and is considered the most damaging and genotoxic, which is chiefly responsible for various sun-induced skin disorders, including sunburn, photoaging, oxidative damage, erythema, acute inflammation, immunosuppression, and nonmelanoma and melanoma skin cancers [3, 4]. UV-B radiation can cause DNA damage as a consequence of its direct action on DNA molecule resulting in the formation of cyclobutane-pyrimidine dimers, 6–4 photoproducts, Dewar isomers and 8-hydroxy-2′-deoxyguanosine, and indirectly increase ROS levels, which, in case of overwhelming the cellular antioxidant defense capacity, leads to oxidative stress and subsequently oxidative photolesions of macromolecules, such as DNA, proteins and lipids, in the skin [5, 6]. ROS participate in diverse UV-B-induced pathophysiological processes by activating mitogen-activated protein kinase (MAPK) and nuclear factor-κB (NF-κB) [7].

Although MMPs play a role in normal tissue development, remodeling and repair, they are closely linked to tissue destruction in a number of pathological conditions, including inflammation, UV-induced skin damage and tumor invasion [8, 9]. MMP-2 and -9, belonging to the gelatinase group of MMPs, degrade the extracellar matrix (ECM) via degrading type IV collagen, the most abundant components of the basement membrane which is important for maintaining tissue organization, providing structural support for cells and influencing cell signaling and polarity, and influence skin wrinkle formation and skin thickness [10]. The exposure of human skin to UV radiation up-regulates the synthesis of various MMPs, including MMP-1, − 2, − 3, − 7, − 8, − 9 and − 12, which are implicated in photoaging [10]. MMP-2 and -9 are expressed at elevated levels in a variety of cell types under oxidative stress [11, 12].

*A. rugosa* is a perennial herb belonging to the mint family (Lamiaceae) cultivated in East Asian Countries, including Korea, and has been used to treat colds, anorexia, cholera, vomiting, miasma and other kinds of disorders [13]. A variety of essential oils, such as methyleugenol, estragole and eugenol, and diverse types of flavonoids, such as tilianin, acacetin, linarin, agastachoside and rosmarinic acid, have been identified from *A. rugosa* [14]. Two diterpenoid compounds, agastanol and agastaquinone, and two lignin compounds, agastinol and agastenol, were also identified from *A. rugosa* [15, 16]. A range of biological and pharmacological activities of *A. rugosa*, including antimicrobial, antifungal, insecticidal, antiviral, antihypertensive, anti-inflammatory, anticancer, antioxidative, antiatherogenic and vasorelaxant activities, have been documented [17–20].

Probiotic bacterial fermentation, emerging as one of crucial processing tools in cosmetic technologies, is used to diminish toxicities of cosmetic resources, enhance absorption into the skin by altering their molecular structures, and improve their desirable pharmacological activities [21].

Throughout the previous work, ARE was reported to have an attenuating potential against the UV-B-induced photoaging of human skin [22]. In the present work, it is demonstrated that the skin anti-photoaging activity of ARE can be potentiated by probiotic bacterial fermentation. This finding might possibly broaden the usefulness of *A. rugosa* in therapeutic as well as cosmetic applications.

## Methods

### Chemicals

Bradford reagent, bovine serum albumin, ascorbic acid (AA), 2,2-azino-bis(3-ethylbenzothiazoline-6-sulfonic acid) (ABTS), ammonium persulfate, 2′,7′-dichlorodihydrofluorescein diacetate (DCFH-DA), 5,5′-dithiobis (2-nitrobenzoic acid) (DTNB), glutathione reductase (GR), catalase, xanthine, xanthine oxidase and NADPH were from Sigma-Aldrich Chemical Co. (St Louis, MO, USA). Cell lysis buffer [25 mM Tris-phosphate (pH 7.8), 2 mM 1,2-diaminocyclohexane-*N*,*N*,*Nv*,*Nv*-tetraacetic acid, 2 mM dithiothreitol, 10% glycerol, 1% Triton X-100] was from Promega Korea (Seoul, Korea).

### Plant material

Dried *A. rugosa* leaves, obtained from a local market in Chuncheon, Korea, were authenticated by Prof. Ki-Oug Yoo (Department of Biological Sciences, College of Natural Sciences, Kangwon National University, Chuncheon, Korea). A voucher specimen (No. KWNU90446) was deposited at the herbarium in the same department.

### Preparation of ARE and ARE-F

As previously described [22], ARE was prepared as follows. Dried *A. rugosa* leaves, ground under liquid nitrogen, were extracted under reflux by placing in a water bath at 90 °C for 4 h. After being filtered through a filter paper, the hot water extract was evaporated to dryness in a freeze dryer, and the extract powder was designated as ARE. The yield was approximately 10.4%.

ARE, resuspended in distilled water, was incubated at 30 °C for 5 days with *L. rhamnosus* HK-9 (KACC 11254P, Korea), centrifuged at 5000 g for 20 min to discard bacterial cells, and concentrated using a freeze dryer to generate fermented extract powder, designated as ARE-F. Prior to the experiments, both ARE and ARE-F were dissolved in dimethyl sulfoxide, and control cells were treated with vehicle only (0.1% dimethyl sulfoxide). The vehicle itself had no damaging effect on the viabilities of HaCaT cells.

### Cell culture

A human immortalized HaCaT keratinocyte cell line (ATCC No. CRL-2309) was obtained from American Type Culture Collection (Manassas, VA, USA) and grown in Dulbecco’s Modified Eagle’s Medium (DMEM) with 10% heat-inactivated fetal bovine serum (FBS), 100 units/mL penicillin and 100 μg/mL streptomycin in a humidified atmosphere with 5% CO_2_ at 37 °C.

### UV-B irradiation

As a UV-B source, an ultraviolet lamp (peak, 312 nm; model VL-6 M, Vilber Lourmat, Marine, France) was used with a radiometer (model VLX-3 W) equipped with a sensor (bandwidth, 280 to 320 nm; model CX-312). In the present work, HaCaT keratinocyte culture at 25 °C were irradiated with solar simulated UV-B radiation at 70 mJ/cm^2^, an intensity chosen to induce a photooxidative stress through a preliminary experiment.

### Preparation of cellular lysate

At 18 h after irradiation, adherent cells, washed twice with PBS and stored on ice for 5 min, were collected using a cell scraper, centrifuged at 5000 g for 10 min, resuspended in cell lysis buffer and stored for 30 min on ice. Cellular lysate was taken out after centrifugation at 5000 g for 15 min.

Protein content in cellular lysates was quantitated with the Bradford protein assay [23] using bovine serum albumin as a reference standard to construct a calibration curve.

### Quantitation of intracellular ROS

As previously described [24], a redox-sensitive fluorescent probe DCFH-DA, which generates the fluorescent 2′,7′-dichlorofluorescein (DCF; λ_excitation_ = 485 nm, λ_emission_ = 530 nm) upon enzymatic reduction and subsequent oxidation by ROS, was utilized to detect intracellular ROS. After HaCaT keratinocytes were treated with ARE and ARE-F and/or 20 μM DCFH-DA for 30 min at 37 °C, they were washed twice with 1 mL FBS-free DMEM. The cells, resuspended in 1 mL FBS-free DMEM, were irradiated with UV-B radiation. Immediately after the irradiation, the intracellular ROS levels were determined by monitoring the DCF fluorescence using a multi-mode microplate reader (Synergy™ Mx, BioTek Instruments, Winooki, VT, USA).

### Western blotting analysis

Western blotting analyses were conducted to detect proMMP-2 and -9 in cellular lysate using anti-MMP-2 (ALX-210-753, Enzo Life Sciences, Farmingdale, NY, USA) and anti-MMP-9 (3852S, Cell Signaling Technology, Danvers, MA, USA) antibodies. Glyceraldehyde 3-phosphate dehydrogenase (GAPDH), used as an internal loading standard, was detected using anti-GAPDH antibody (LF-PA0212, AbFrontier, Seoul, Korea). Cellular lysates were separated on 10% (*w*/*v*) SDS-PAGE and electrotransferred to PVDF membrane. After the blotted membrane was probed with primary antibodies overnight at 4 °C, it was incubated with secondary antibody (goat anti-rabbit IgG-pAb-HRP-conjugate; ADI-SAB-300, Enzo Life Sciences, Farmingdale, NY, USA) for 1 h at room temperature, and developed using an enhanced West-save up™ (AbFrontier, Seoul, Korea).

### Determination of total GSH

Total GSH content in cellular lysates was determined using an enzymatic recycling assay based on GR [25]. The reaction mixture (200 μL), which contained 175 mM KH_2_PO_4_, 6.3 mM EDTA, 0.21 mM NADPH, 0.6 mM DTNB, 0.5 units/mL GR and cellular lysate, was incubated at 25 °C. A change in absorbance at 412 nm was monitored using a microplate reader. Total GSH contents were calculated from a calibration curve constructed with various GSH concentrations, and normalized to the total protein content of cellular lysates, which gave rise to total GSH content in μg/mg protein.

### Determination of SOD activity

Total SOD activity in cellular lysates was determined as the reduction of cytochrome c with xanthine/xanthine oxidase system [26]. The reaction mixture (200 μL), contained 50 mM phosphate buffer (pH 7.4), 0.01 units/mL xanthine oxidase, 0.1 mM EDTA, 1 μM catalase, 0.05 mM xanthine, 20 μM cytochrome c and cellular lysate. A change in absorbance at 550 nm was monitored using a microplate reader. The SOD activity was normalized by determining the protein content of cellular lysates, and represented as ΔA_550_/min/mg protein.

### Quantitation of antiradical activity

Antiradical activities of ARE and ARE-F were detected using ABTS radical scavenging assay [27] with a slight modification. ABTS radical cations (ABTS^+^), produced by reacting ABTS stock solution (0.07 mM) with 0.12 mM ammonium persulfate, were kept to stand in the dark at room temperature for 16 h before use. The varying concentrations of ARE and ARE-F (each 10 μL) were mixed with 290 μL of ABTS^+^ solution and the final volume was made up to 1 mL with ethanol. The reaction mixture was incubated for 15 min in the dark at room temperature. AA was used as a positive control. The absorbance was measured at 745 nm and the percent inhibition by ARE and ARE-F was calculated using the formula, Inhibition (%) = [(Control - Test)/Control] × 100. The AA concentration eliciting 50% scavenging of ABTS^+^ radicals (SC_50_) was calculated.

### Statistical analysis

The data were expressed as mean ± SD. Differences between experimental groups were analyzed using one-way ANOVA followed by post hoc Tukey HSD test for multiple comparisons. A *P* value < 0.05 was considered statistically significant.

## Results

### Reactive oxygen species (ROS)

ROS are generated by exogenous (environmental) sources, such as UV irradiation and heat exposure, in addition to their production as natural byproducts of the normal cellular metabolism of oxygen. Oxidative stress, which refers to the excessively elevated intracellular levels of ROS and participate in a myriad of pathologies, arises from an imbalance between the production of ROS and the cellular capability to scavenge the reactive intermediates and to repair the resulting damage. ROS levels, one of principal markers of oxidative stress, are considered to reflect the degree of oxidative stress.

**Fig. 1:**
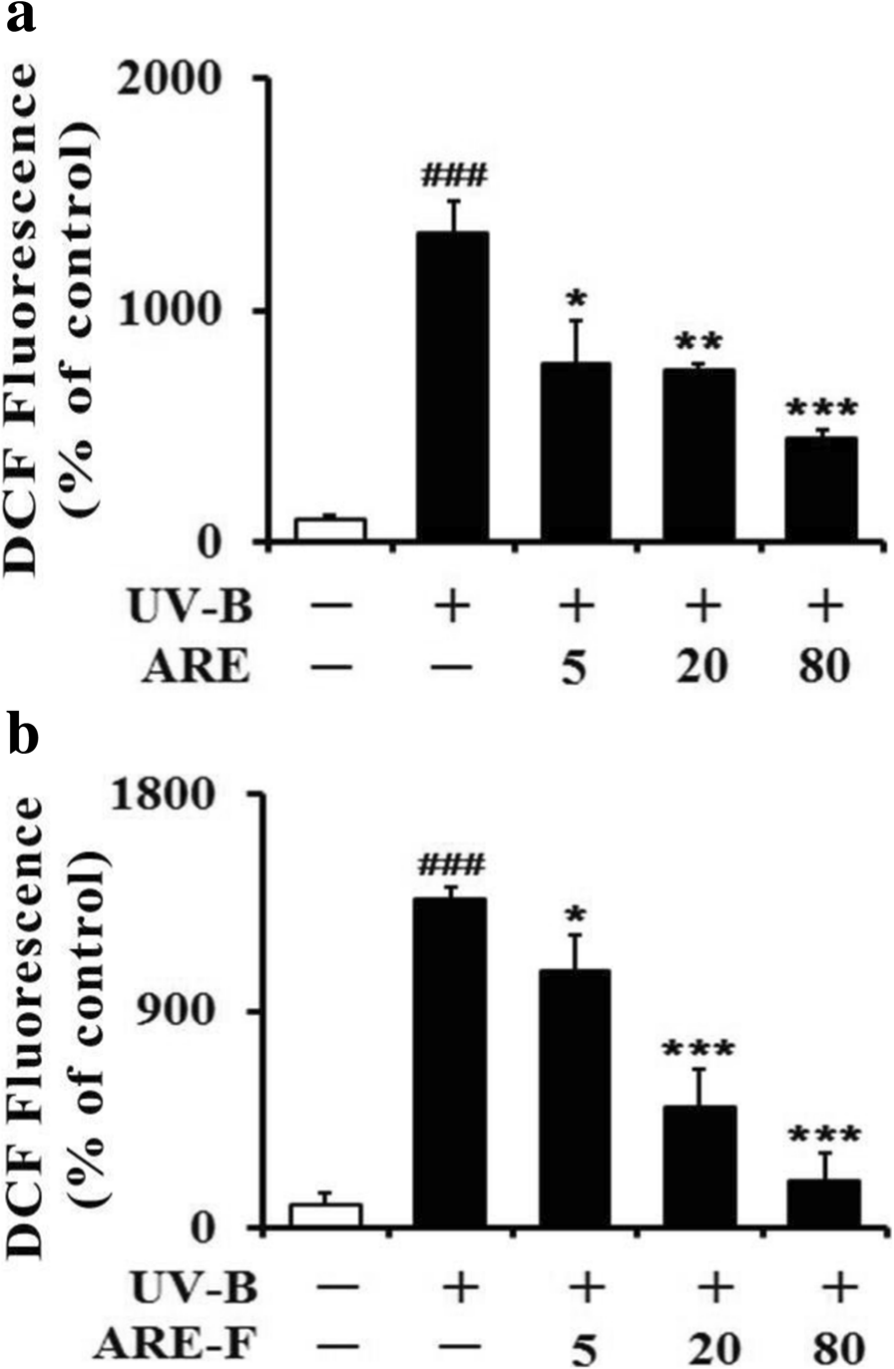
Suppressive effects of ARE, (**a**) and ARE-F (**b**) on the ROS levels in HaCaT keratinocytes under UV-B irradiation. HaCaT cells were subjected to the varying concentrations (0, 5, 20 and 80 μg/mL) of ARE (**a**) and ARE-F (**b**) for 1 h before the irradiation. The ROS levels were determined using DCFH-DA in a microplate fluorometer, and represented as DCF fluorescence, expressed as a percentage (%) of the corresponding non-irradiated control. ^###^*P* < 0.001 versus the non-irradiated control. *P < 0.05; **P < 0.01; ***P < 0.001 versus the non-treated control (UV-B irradiation alone)

When HaCaT keratinocytes were irradiated with 70 mJ/cm^2^ UV-B radiation, the intracellular ROS levels went up to approximately 15.3-fold (Fig. 1). This result could prove that UV-B irradiation, at the intensity chosen in this work, can impose a state of oxidative stress on HaCaT keratinocytes. However, the radiation intensity used couldn’t interfere with the viabilities of HaCaT keratinocytes, which was detected using an MTT assay (data not shown). When HaCaT keratinocytes were subjected to varying concentrations (5, 20 and 80 μg/mL) of ARE prior to the irradiation, ARE attenuated the UV-B-induced ROS levels to. 57.4, 55.7 and 32.8%, respectively (Fig. 1a). In the same pretreatment, ARE-F, at the concentrations of 5, 20 or 80 μg/mL, could attenuate the UV-B-induced ROS levels to 60.7, 36.1 and 13.1%, respectively (Fig. 1b). ARE exhibited an IC_50_ value of 27.0 μg/mL, whereas that of ARE-F was determined to be 17.4 μg/mL. Collectively, ARE-F has an enhanced attenuating activity on the UV-B-induced ROS levels than ARE, implying that the intracellular antioxidative activity of ARE can be augmented by probiotic bacterial fermentation.

### Promatrix metalloprtoenase-9 (proMMP-9) and − 2 (proMMP-2)

**Fig. 2:**
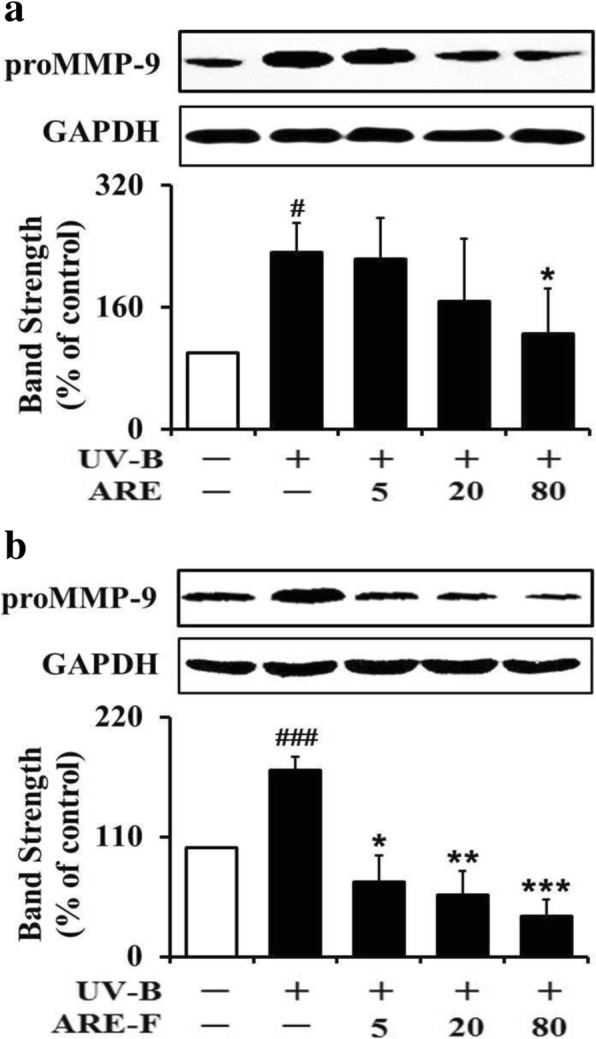
Suppressive effects of ARE (**a**) and ARE-F (**b**) on the proMMP-9 elevation in HaCaT keratinocytes under UV-B irradiation. HaCaT cells were subjected to the varying concentrations (0, 2, 20 and 80 μg/mL) of ARE (**a**) and ARE-F (**b**) for 1 h before the irradiation. The proMMP-9 proteins in cellular lysates, detected using western blotting analysis, were expressed as a percentage (%) of the corresponding non-irradiated control. In the lower panels of both **a** and **b**, the band strength was determined with densitometry using the ImageJ software which can be downloaded from the NIH website. ^#^*P* < 0.05; ^###^*P* < 0.001 versus the non-irradiated control. *P < 0.05; **P < 0.01; ***P < 0.001 versus the non-treated control (UV-B irradiation alone)

The skin photoaging activity of UV-B radiation is mediated by the enhancement of diverse MMPs by UV-B-induced ROS. MMP-1, − 9 and − 2, known to be directly involved in the impairment of ECM components, were shown to be up-regulated by UV-B radiation [10]. Even the precursor forms (proMMP-9 and -2) of MMP-9 and -2 were previously identified to be enhanced in HaCaT keratinocytes by UV-B irradiation [28]. As shown in Fig. 2, the UV-B irradiation alone could give rise to approximately 2.0-fold elevation in the proMMP-9 protein level over that in the non-irradiated control cells. HaCaT keratinocytes were subjected to varying concentrations (0, 5, 20 and 80 μg/mL) of ARE prior to UV-B irradiation. ARE at the concentrations of 5, 20 and 80 μg/mL made the UV-B-induced proMMP-9 elevation reduce to 96.1, 72.5 and 54.9% of that from the UV-B irradiation alone, respectively (Fig. 2a). As shown in Fig. 2b, the attenuating activity of ARE-F was also examined in a similar way. ARE-F at the concentrations of 5, 20 and 80 μg/mL was able to attenuate the UV-B-induced proMMP-9 elevation to 39.6, 32.1 and 20.8%, respectively (Fig. 2b). The IC_50_ values of ARE and ARE-F in the attenuating activities on proMMP-9 were 91.8 and 11.2 μg/mL, respectively. In brief, ARE-F has higher attenuating activity on the UV-B-induced proMMP-9 elevation than ARE.

**Fig. 3:**
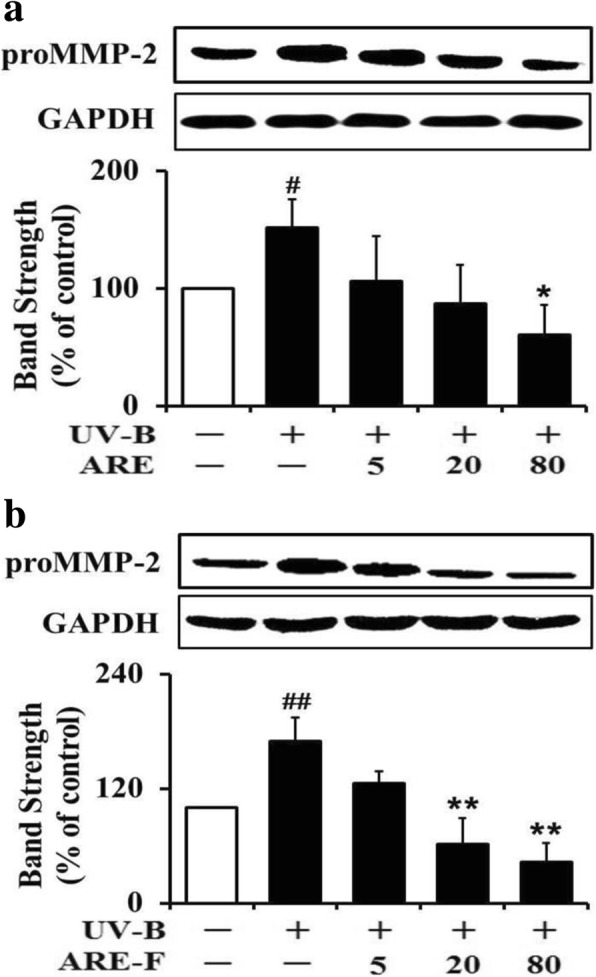
Suppressive effects of ARE (**a**) and ARE-F (**b**) on the proMMP-2 elevation in HaCaT keratinocytes under UV-B irradiation. HaCaT cells were subjected to the varying concentrations (0, 5, 20 and 80 μg/mL) of ARE (**a**) and ARE-F (**b**) for 1 h before the irradiation. The proMMP-2 proteins in cellular lysates, detected using western blotting analysis, were expressed as a percentage (%) of the corresponding non-irradiated control. In the lower panels of both **a** and **b**, the band strength was determined with densitometry using the ImageJ software which can be downloaded from the NIH website. ^#^*P* < 0.05; ^##^*P* < 0.01 versus the non-irradiated control. *P < 0.05; **P < 0.01 versus the non-treated control (UV-B irradiation alone)

As shown in Fig. 3, the attenuating activities of both ARE and ARE-F on the UV-B-induced proMMP-2 levels were also measured and compared. The UV-B irradiation alone induced the proMMP-2 protein levels to about 1.7-fold over the non-irradiated control value (Fig. 3). ARE at the concentrations of 5, 20 and 80 μg/mL attenuated the UV-B-induced proMMP-2 protein to 69.5, 57.6 and 39.0% of that of the UV-B irradiation only (Fig. 3a). In an analogous experiment, ARE-F at the concentrations of 5, 20 and 80 μg/mL could attenuate the UV-B-induced proMMP-2 protein levels to 74.1, 37.0 and 26.0%, respectively, of that of the UV-B irradiation only (Fig. 3b). The IC_50_ values of ARE and ARE-F were determined to be 48.3 and 27.9 μg/mL. Taken together, the proMMP-2-downregulating activity of ARE is also enhanced by probiotic fermentation.

### Total glutathione (GSH)

**Fig. 4:**
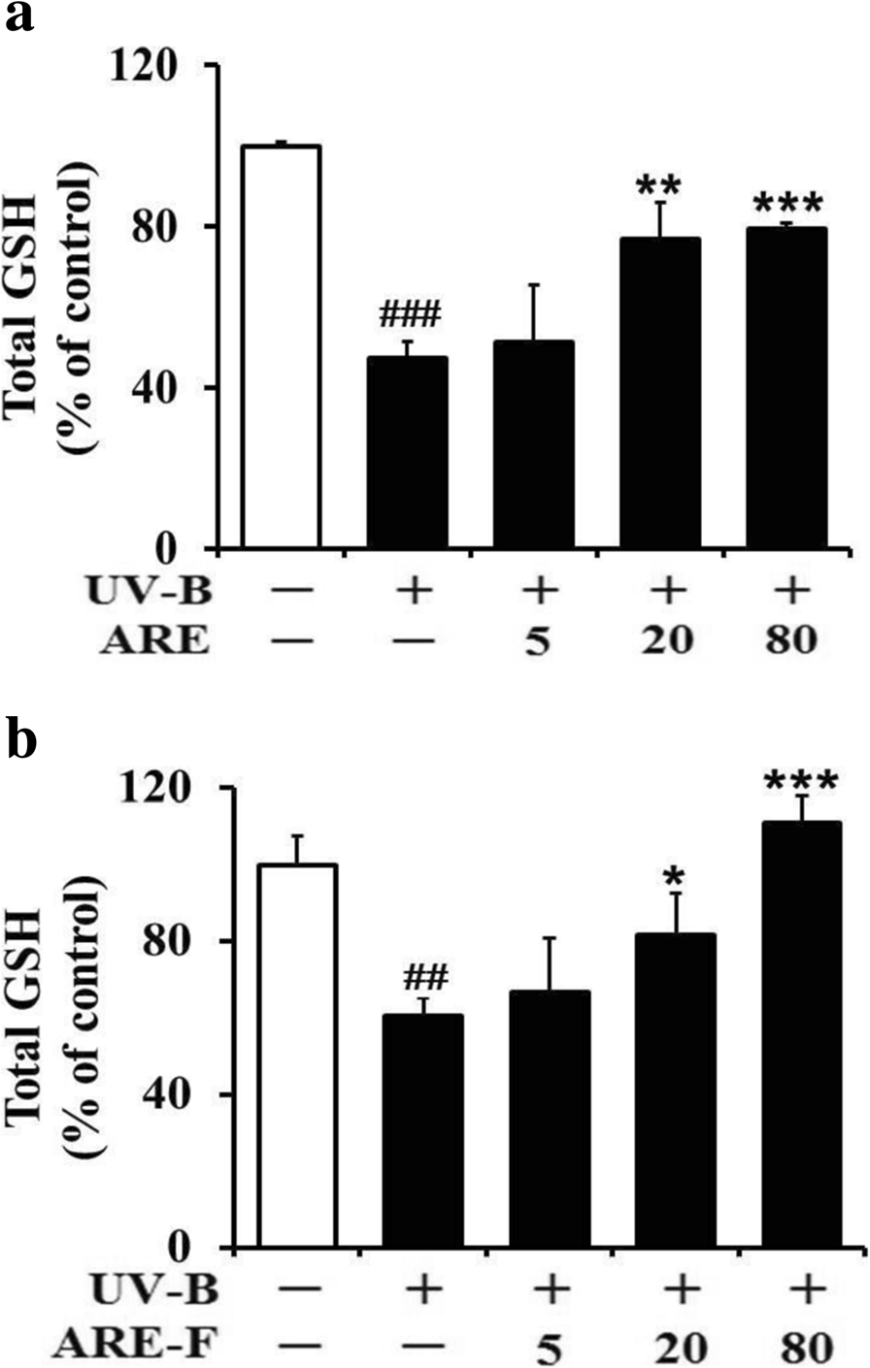
Enhancing effects of ARE (**a**) and ARE-F (**b**) on the total GSH attenuation in HaCaT keratinocytes under UV-B irradiation. HaCaT cells were subjected to the varying concentrations (0, 5, 20 and 80 μg/mL) of ARE (**a**) and ARE-F (**b**) for 1 h before the irradiation. The total GSH content in the cellular lysates, quantitated with an enzymatic recycling assay using GR, was expressed as a percentage (%) of the corresponding non-irradiated control. ^##^*P* < 0.01; ^###^*P* < 0.001 versus the non-irradiated control. *P < 0.05; **P < 0.01; ***P < 0.001 versus the non-treated control (UV-B irradiation alone)

GSH plays a protective property against oxidative stress in various cell types, including skin cells and has its protective property against oxidative stress via directly or indirectly scavenging ROS. In the previous work, total GSH, including oxidized and reduced GSH, was shown to decline in UV-B-irradiated keratinocytes [29]. Likewise, total GSH levels were significantly decreased in HaCaT keratinocytes under UV-B irradiation, compared to the non-irradiated control value (Fig. 4). ARE at the concentrations of 5, 20 and 80 μg/mL enhanced the UV-B-reduced total GSH levels to 1.1-, 1.6- and 1.7-fold of the non-treated value, respectively (Fig. 4a). Similarly, ARE-F at the concentrations of 5, 20 and 80 μg/mL was able to enhance the UV-B-reduced total GSH levels to 1.1-, 1.4- and 1.9-fold of the non-treated value, respectively (Fig. 4b). ARE and ARE-F at 80 μg/mL could restore the UV-B-reduced total GSH levels to 80.0 and 111.2%, respectively, of the non-irradiated value (Fig. 4). This finding implies that the GSH-restoring activity of ARE on the UV-B-reduced total GSH levels is enhanced by probiotic fermentation.

### Total superoxide dismutase (SOD)

**Fig. 5:**
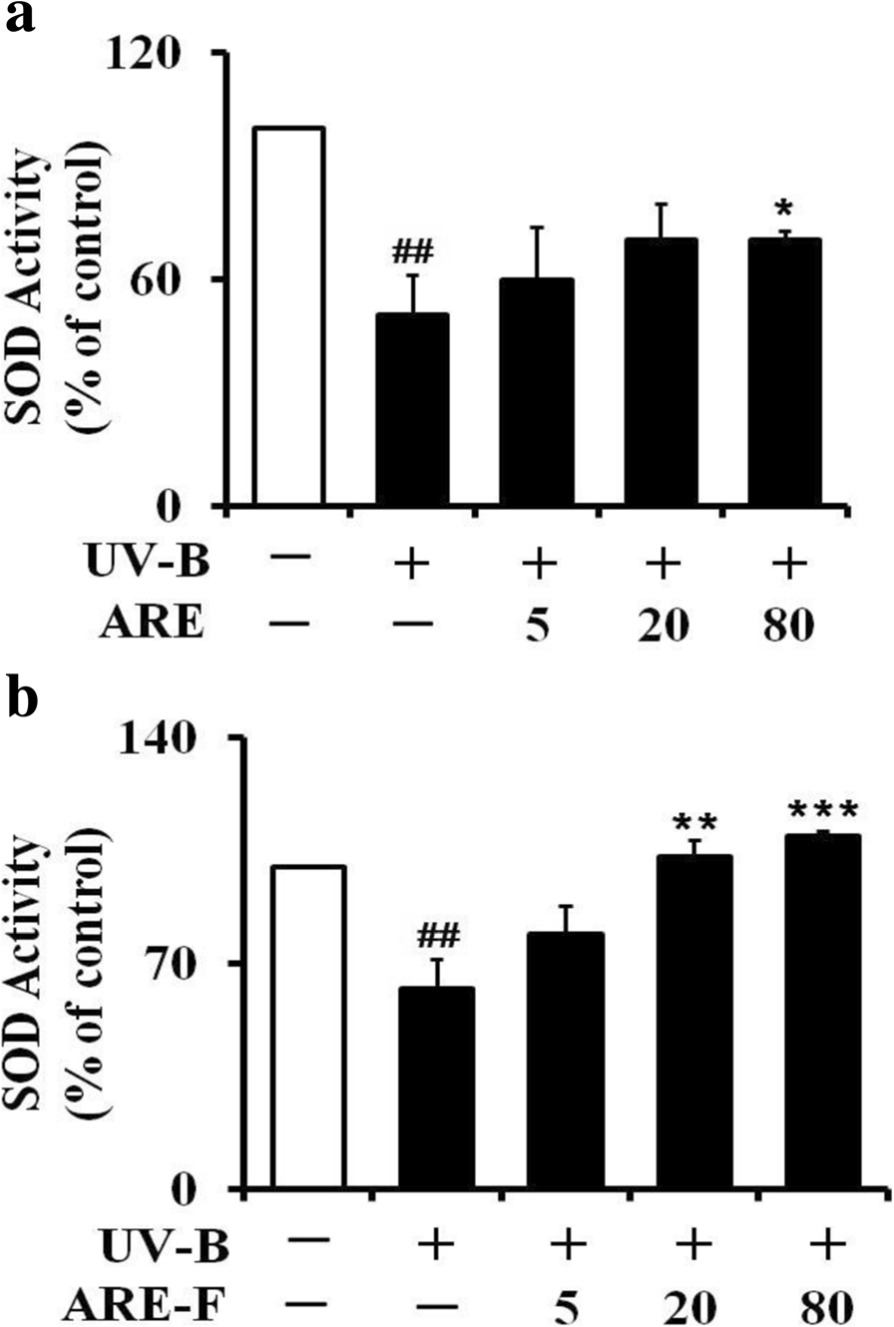
Enhancing effects of ARE (**a**) and ARE-F (**b**) on the total SOD activity attenuation in HaCaT keratinocytes under UV-B irradiation. HaCaT cells were subjected to the varying concentrations (0, 5, 20 and 80 μg/mL) of ARE (**a**) and ARE-F (**b**) for 1 h before the irradiation. The total SOD activity in the cellular lysates, expressed as a percentage (%) of the corresponding non-irradiated control, was determined using a spectrophotometric assay. ^##^*P* < 0.01 versus the non-irradiated control. *P < 0.05; **P < 0.01; ***P < 0.001 versus the non-treated control (UV-B irradiation alone)

SOD, deeply involved in the defensive mechanisms against oxidative stress, was previously identified to decline in UV-B-irradiated keratinocytes [30]. In this work, UV-B irradiation markedly diminished the total SOD activity, compared to the non-irradiated value (Fig. 5). As shown in Fig. 5a, ARE at the concentrations of 5, 20 and 80 μg/mL enhanced the UV-B-reduced SOD activity to 1.3-, 1.4 and 1.4-fold over that of the UV-B irradiation only. ARE-F at the concentrations of 5, 20 and 80 μg/mL was able to increase the UV-B-reduced total SOD activity to 1.1-, 1.6- and 1.8-fold, respectively, over that of the UV-B irradiation only (Fig. 5b). ARE and ARE-F at the concentration of 80 μg/mL could restore the UV-B-reduced total SOD activity levels to 70.4 and 110.2%, respectively, over that of the non-irradiation only (Fig. 5). Taken together, the SOD-restoring activity of ARE is up-regulated by probiotic fermentation.

### In vitro antiradical activity

**Fig. 6:**
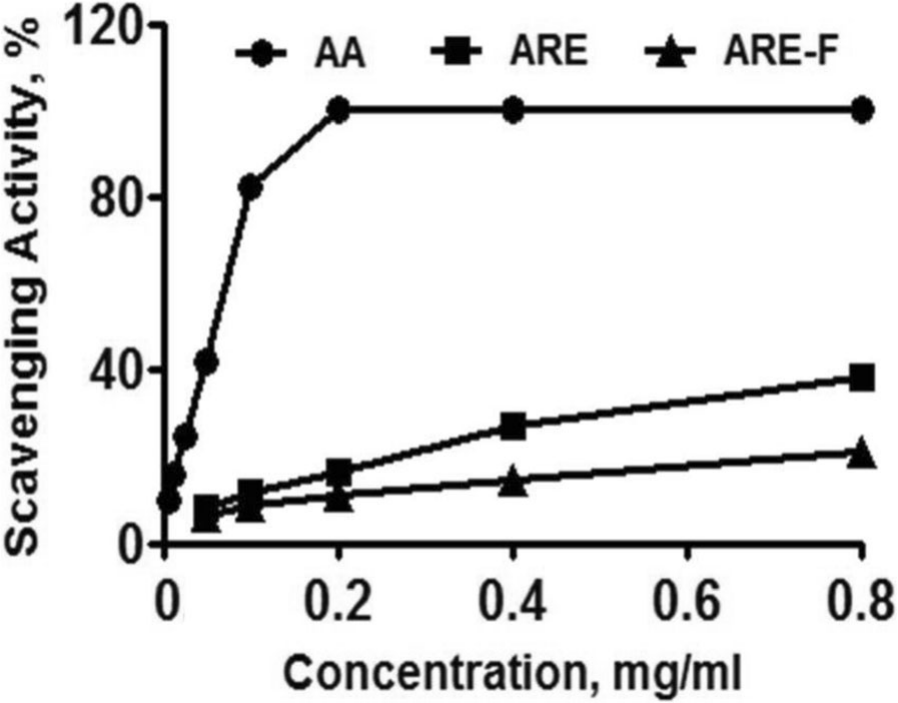
The ABTS radical scavenging activities of ARE and ARE-F. AA was used as a positive control. Each value shows the mean ± SD of the three independent experiments repeated in triplicate

In order to compare the antiradical activities of ARE and ARE-F, the ABTS radical scavenging assay was conducted. AA, used as a positive control, was found to display an SC_50_ of 49.0 μg/mL (Fig. 6). Both ARE and ARE-F exhibited relatively weak ABTS radical scavenging activities, compared to AA and they didn’t display a significant difference in ABTS radical scavenging activities (Fig. 6). This result suggests that the potentiation of the skin anti-photoaging properties of ARE by probiotic fermentation might not be irrespective of a change in in vitro antiradical activity.

## Discussion

Through the recent years, the biological and pharmacological efficacies of *A. rugosa* have been continuously and extensively studied. An extract of *A. rugosa* leaves protects RAW264.7 cells from hydrogen peroxide-induced injury via the induction of protein kinase G-dependent heme oxygenase-1 [31]. Essential oils of *A. rugosa* leaves, whose major compounds are *p*-menthan-3-one and estragole, have antimicrobial, antifilm and antitumor activities [32]. Demethyleugenol β-glucopyranoside, isolated from *A. rugosa*, possesses has an ameliorating activity on skin pigmentation by decreasing melanin synthesis via down-regulating microphthalmia-associated transcription factor and sex-determinating region Y-box 9 and subsequently resulting in a decrease in melanogenic enzymes, such as tyrosinase, tyrosinase-related protein 1 and dopachrome tautomerase [33]. An ethanol extract of *A. rugosa* leaves has an antimelanogenic activity through the suppression of tyrosinase, which is enhanced by the fermentation with *L. rhamnosus* and *L. paracasei* [34]. Addition of yeast extract, as an elicitor, to the *A. rugosa* culture enhances the accumulation of rosmarinic acid, a main phenylpropanoid of *A. rugosa*, through the up-regulation of phenylpropanoid biosynthetic pathway genes, such as hydroxyl phenylpyruvate reductase and rosmarinic acid synthase genes [35]. A hot water extract of *A. rugosa* leaves have skin anti-photoaging property against UV-B-induced photooxidative stress in human keratinocytes via up-regulating GSH and SOD [22]. These findings further support the valuable application potential of *A. rugosa* in the manufacture of functional cosmetics. Enhancement of the desirable efficacies of *A. rugosa* would make its application more convenient and more economical.

Since useful phytochemicals, as nature’s chemicals, are believed to be more safe than synthetic chemicals, they increasingly attract great interest in the healthcare, food, flavor and cosmetics industries. Fermentation of phytochemicals is a crucial processing method and is attracting a lot of attention since it may have the advantage of having novel and augmented biological functions. Desirable cosmetic ingredients need to contain attractive properties, including antioxidant, anti-inflammatory, skin whitening, anti-aging, moisturizing, bio-anti-wrinkle activities and so on [36]. During fermentation processing, microbes, including lactic acid bacteria, can improve the cellular functions of plant materials through their enzymatic activities, which promotes the production of a variety of metabolites and/or the release of functional components that are cryptic in non-fermented materials [37]. Bioconversion using whole cells is usually more stable, inexpensive and convenient than that using a purified enzyme [38]. Some biosurfactant extracts from Chinese medical herb fermentation exhibit favorable antioxidative, emulsifying and moisturizing properties in cosmetic formulations [39].

Fermentation with lactic acid bacteria is one of the most valuable tools to exploit the desirable functions of plant resources and to enrich them with bioactive compounds [40]. Fermentation with several lactic acid bacteria has been used to improve the antimicrobial, antioxidant and immunomodulatory activities of natural compounds of plant and microbial origins [41]. Phenolic compounds are well-known secondary metabolites of various plants that have been subjected to the lactic acid bacteria fermentation for studying the accompanying changes [42]. Bioconversion of baicalin and wogonoside to baicalein and wogonin, two main flavonoids exhibiting increased beneficial pharmacological properties in *Scutellaria baicalensis*, is significantly enhanced during fermentation using β-glucuronidase from *L. brevis* RO1 [43]. Myrtle berries homogenate, fermented with *L. plantarum* C2, was found to contain the increased antioxidant activity, probably based up on the increased concentrations of total phenols (mainly, gallic acid and ellagic acid), anthocyanins and flavonoids (mainly, myricetin and quercetin) [44]. Dried funori, a non-toxic, all-natural starch derived from seaweed (*Gloiopeltis furcata* and *Gloiopeltis tenax*), displays a significant enhancement in the superoxide anion radical-scavenging capacity after the fermentation with *L. plantarum* S-SU1 [45]. Fermentation of *Psidium guajava* fruit extract using *L. plantarum* NCIM 2912 enhances its antioxidant potential as well as total phenolics and short and medium chain fatty acid contents [46]. Fermenting a red ginseng extract with *L. brevis* enhances contents of ginsenoside metabolites, such as Rg3, Rg5, Rk1, compound K, Rh1, F2 Rg2, uronic acid, polyphenols and flavonoids, and subsequently offers increased anti-wrinkle and whitening efficacies and diminished toxicological potency [47]. *L. plantarum*– and *Bifidobacterium bifidum*-fermented aqueous extracts of *Acanthopanax koreanum* roots exhibit enhanced antioxidant and antisenescent activities against the exposure to UV-B irradiation and hydrogen peroxide, implying the improved anti-wrinkle effect on human skin [21]. The aloe fermentation supernatant fermented by *L. plantarum* possess enhanced antioxidant, antibacterial and anti-inflammatory activities [48]. In this work, we demonstrate that the skin anti-photoaging properties of a hot water extract of *A. rugosa* leaves are significantly augmented by the fermentation with *L. rhamnosus* HK-9, although the underlying mechanism currently remains uncertain. However, this result may elevate its application potential in cosmetics industries.

In this work, ARE-F tended to exhibit a diminished ABTS radical scavenging activity, compared to ARE, which might be contrary to its intracellular defensive properties in HaCaT keratinocytes. Although the cause of this discrepancy remains to be clarified, some findings on the reduction of antioxidant components and activities during fermentation were previously reported. The fermentation of strawberry must with *Saccharomyces cerevisiae* was found to diminish the ABTS and 2,2-diphenyl-1-picrylhydrazyl (DPPH) radical scavenging activities [49]. The liquid state fermentations of in vitro sprout and shoot cultures of java tea with *L*. *acidophilus* and *L. plantarum* cause a significant reduction in the levels of rosmarinic acid and total phenolic compounds and a loss of antioxidant activities, such as DPPH and ABTS scavenging activities and SOD-like activity [50, 51]. In these experiments, the degree of reduction in some antioxidant components and activities was found to depend on the fermentation parameters, such as probiotic strains, fermentation temperature and fermentation period. Further approaches in future would help understand the discrepancy obtained in this work.

## Conclusions

In conclusion, the present work demonstrates that the fermentation of *A. rugosa* leaf extract with a probiotic *Lactobacillus* strain improves its skin anti-photoaging properties through further augmenting UV-B-reduced total GSH and SOD activity levels and increasingly attenuating UV-B-induced ROS and MMP-2 and -9 levels in some sequential order. These findings imply the potential augmentation of the skin anti-photoaging properties of *A. rugosa* by probiotic fermentation, which can expand its usefulness in various applications, including cosmetic manufacture.

## Abbreviations

AA: ascorbic acid
ABTS: 2,2-azino-bis(3-ethylbenzothiazoline-6-sulfonic acid)
ARE: a non-fermented hot water extract of *A. rugosa* leaves
ARE-F: a fermented hot water extract of *A. rugosa* leaves
DCF, 2′,7′-dichlorofluorescein: DCFH-DA, 2′,7′-dichlorodihydrofluorescein diacetate
DMEM: Dulbecco’s modified Eagle’s medium
DNTB: 5,5′-dithiobis (2-nitrobenzoic acid)
FBS: fetal bovine serum
GSH: glutathione
GAPDH: glyceraldehyde 3-phosphate dehydrogenase
GR: Glutathione reductase
IC_50_: 50% inhibitory concentration
MMP: Matrix metalloproteinase
PBS: Phosphate-buffered saline
proMMP: promatrix metalloproteinase
ROS: Reactive oxygen species
SC_50_: 50% scavenging concentration
SOD: Superoxide dismutase
UV-B: Ultraviolet-B
